# Enhanced surveillance of invasive listeriosis in the Lombardy region, Italy, in the years 2006-2010 reveals major clones and an increase in serotype 1/2a

**DOI:** 10.1186/1471-2334-13-152

**Published:** 2013-03-26

**Authors:** Caterina Mammina, Antonio Parisi, Anna Guaita, Aurora Aleo, Celestino Bonura, Antonino Nastasi, Mirella Pontello

**Affiliations:** 1 Department of Sciences for Health Promotion “G. D’Alessandro”, University of Palermo, Palermo, Italy; 2 Experimental Zooprophylactic Institute of Apulia and Basilicata, Foggia, Italy; 3 Department of Sciences for Health, University of Milan, Milan, Italy; 4 Department of Public Health, University of Florence, Florence, Italy

## Abstract

**Background:**

Invasive listeriosis is a rare, life-threatening foodborne disease. Lombardy, an Italian region accounting for 16% of the total population, reported 55% of all listeriosis cases in the years 2006-2010. The aim of our study was to provide a snapshot of listeriosis epidemiology in this region after the implementation of a voluntary laboratory-based surveillance system.

**Methods:**

We characterized by serotyping, pulsed-field gel electrophoresis, multilocus sequence typing and detection of epidemic clone markers, 134 isolates from 132 listeriosis cases, including 15 pregnancy-related cases, occurring in the years 2006-2010 in Lombardy. Demographic and clinical characteristics of cases have also been described.

**Results:**

The mean age of non pregnancy-associated cases was 64.7 years, with 55.9% of cases being older than 65 years. Cases having no underlying medical conditions accounted for 11.6%. The all-cause fatality rate of 83 cases with a known survival outcome was 25.3%.

Serotypes 1/2a and 4b comprised 52.2% and 38.8% of isolates, respectively. Seventy-three *Asc*I pulsotypes and 25 sequence types assigned to 23 clonal complexes were recognized. Moreover, 53 (39.5%) isolates tested positive for the epidemic clone markers. Twelve molecular subtype clusters including at least three isolates were detected, with cluster 11 (1/2a/ST38) including 31 isolates identified during the entire study period. No outbreaks were notified to public health authorities during this period.

**Conclusions:**

The findings of our study proved that epidemiology of listeriosis in Lombardy is characterized by a high prevalence of major clones and the increasing role of serotype 1/2a. Molecular subtyping is an essential tool in the epidemiology and surveillance of listeriosis. Rapid molecular cluster detection could alert about putative outbreaks, thus increasing the chance of detecting and inactivating routes of transmission.

## Background

Invasive listeriosis is an infrequent, life-threatening disease. Its case-fatality rate ranges between 20 and 50%, accounting for 30% approximately of all foodborne disease associated deaths [1-4]. Immunocompromised patients are at high risk of invasive listeriosis, which can evolve into severe forms of septicaemia, meningoencephalitis and death [1-4]. Pregnant women more typically experience mild illness or no symptoms at all, but fetal and neonatal infections are severe and frequently fatal [4,5].

Increasing incidence rates of invasive listeriosis have been reported in the last decade by several European countries [6-9]. However, the European Food Safety Authority (EFSA)/European Centre for Disease Prevention and Control (ECDC) report on the occurrence of foodborne diseases shows an overall EU notification rate of 0.35 cases per 100,000 population in 2010 with a 3.2% decrease compared with 2009 [10].

In Italy, notification of listeriosis has been mandatory since 1993. A second national syndrome-based surveillance system, covering infections of the central nervous system, is in place. A national reference laboratory, which receives strains and epidemiological and clinical information on a voluntary basis, is also present. The notification rate for confirmed cases in the years 2006-2010 was lower than most EU countries, being equal to 0.16 per 100,000 population [10].

Between 1991 and 2002, in Europe, a total of 19 outbreaks of invasive listeriosis have been notified in nine different countries, with a total of 526 outbreak-related cases [11]. Human infection occurs by consumption of contaminated foods, but the heterogeneity of food products, the extended shelf life of many marketed products and the prolonged incubation period jeopardize the identification of common source outbreaks and food vehicles.

Discriminative molecular subtyping methods are necessary for timely outbreak detection and tracing of contamination sources. Indeed, phenotypic methods, such as serotyping, have low discriminatory power and poorly support epidemiological investigations and surveillance [12]. Although alternative subtyping systems are increasingly used, pulsed-field gel electrophoresis (PFGE) is still considered the standard subtyping method for *L. monocytogenes* due to its high reproducibility and discriminatory power [12,13]. Multilocus sequence typing (MLST) has also been used for some years, the most important advantage being the unambiguity and electronic portability of subtyping data [14].

By molecular subtyping methods seven epidemic clones (ECs) of *L. monocytogenes* have been characterized. In particular, ECI and ECIV appear to be cosmopolitan clones [15,16]. ECII has been associated with multistate outbreaks in 1998 to 1999 and in 2002 in the United States, and has been previously described in Italy [17]. ECIII isolates have been linked to a 1988 sporadic listeriosis case and a multi-state outbreak that occurred in United States during 2000 [16]. ECV has been recently proposed as the predominant clone causing human sporadic and outbreak-related cases in Canada between 1988 and 2010 [18]. Finally, ECVI and ECVII have been identified during the investigation of a foodborne outbreak associated with cantaloupe consumption occurring in 2011 in the United States [19].

This study is a retrospective analysis of 134 human isolates of *L. monocytogenes* identified from apparently sporadic cases of infection in the Lombardy region, Italy, in the years 2006-2010. During the study period, this region accounted approximately for 16% of the Italian population, but for 55% of the notified listeriosis cases in the entire country [20]. The objectives were to assess clonality of *L. monocytogenes* isolates by using serotyping, PFGE and MLST and to evaluate the distribution of EC markers by multiplex PCR and multi-virulence-locus sequence typing (MVLST). Some demographic and clinical features of the listeriosis cases are also described.

## Methods

### Data source and listeriosis case definition

In Lombardy, the national mandatory notification system has since 2005 been integrated with a laboratory-based network involving voluntary referral of clinical isolates to the regional reference laboratory at the University of Milan. This laboratory carries out serotyping and molecular subtyping to detect clusters and support epidemiological investigations. Moreover, by using a standardized report form, demographic (e.g. age, gender, province of residence), clinical (e.g. symptoms, clinical form of disease, existence of underlying conditions, patient outcome) and microbiological (type and number of the positive biological samples) data are routinely collected.

For the purposes of the study, a listeriosis case was defined as isolation of *L. monocytogenes* from a normally sterile site [e.g. blood or positive cerebrospinal fluid (CSF)] or from products of conception. Cases were classified as non pregnancy-associated listeriosis and pregnancy-associated listeriosis, the latter comprising those occurring in pregnant women or in infant aged less than six weeks. Each mother-infant pair was counted as a single case. Clinical forms of disease were categorized as septicemia, meningitis (including cases with concomitant septicemia), and other infections. Underlying conditions were defined as conditions preceding the listeriosis onset based on a retrospective medical chart review.

### Isolate characterization

All human *L. monocytogenes* isolates collected by the regional reference laboratory of Lombardy at the University of Milan from 2006 to 2010 were studied. The strains had been isolated and identified by standard methods in the routine clinical microbiology laboratories of the healthcare facilities of the Lombardy region.

All isolates were serotyped by using antisera against O and H antigens according to the instructions of the manufacturer (Denka Seiken Co., Ltd, Tokyo, Japan).

PFGE was performed according to the PulseNet protocol with the *Asc*I and *Apa*I enzymes [13]. *Xba*I-digested DNA from *Salmonella enterica* serotype Braenderup H9812 was used as the size reference standard. After *Asc*I digestion, similarity clustering analysis was performed with BioNumerics software (version 5.1; Applied Maths, Saint-Martins-Latem, Belgium) by using the unweighted pair group-matching algorithm and the Dice correlation coefficient with a tolerance of 1.5% [13]. When the similarity value was over 80%, two PFGE types were regarded as being closely related. Indistinguishable or closely related strains were subsequently restricted with *Apa*I for clustering confirmation.

Sequence types (STs) were identified by PCR amplification using previously described primers, with the exception of the *ldh* gene for which primers from a modified MLST scheme [http://www.pasteur.fr/mlst] were used [14,21]. Alleles and STs were assigned by submitting the DNA sequences to the *Listeria* MLST database at the Pasteur Institute, France (http://www.pasteur.fr/mlst). A comparison of STs was performed by the minimum spanning tree algorithm in the Bionumerics software. Strains were grouped into clonal complexes (CCs), defined as groups of profiles differing by no more than one gene from at least one other profile of the group using the entire *Listeria* MLST database [22].

ECs I, II, III and V were first detected by the multiplex PCR protocol described by Chen and Knabel [23], modified according to Knabel *et al.*[18]. Subsequently, detection of ECIV and confirmation of the previous PCR findings for ECI, II, III and V were performed by MVLST [24].

### Statistical methods

The descriptive analysis was performed by calculating the means ± standard deviation and frequencies. The significance of differences was assessed by one-way ANOVA test or Kruskall-Wallis, when appropriate, or by the exact test of Fisher, respectively (Epi-Info 6.04 software, Centers for Disease Control and Prevention, Atlanta, GA, US). A *p* value of ≤0.05 indicated statistical significance.

The study was approved by the Ethics Committee of the University of Milan.

## Results

During the five-year period under study, 134 isolates of *L. monocytogenes* were collected from 132 cases of invasive listeriosis, accounting for 67.3% of the cases notified to the regional health service during same period. *L. monocytogenes* isolations ranged between 18 and 26 in the years 2006-2009 and peaked in 2010, when they rose up to 47.

Fifteen cases were pregnancy-associated (in one case isolates from both mother and child were included) and 118 non pregnancy-associated (Table 1). In this latter group the mean age of patients was 64.7 ± 15.1 years, with 55.9% of cases being older than 65 years. Within these cases, the mean age of the septicemia and meningitis cases was similar (65.5 ± 14.9 vs. 63.2 ± 16.2 years, P = 0.63). Out of 118 isolates from non pregnancy-associated cases, 114 (96.6%) were isolated from blood and/or CSF. Cases having no underlying medical conditions were 12 (11.6%) out of 103 with a known clinical history. The all-cause fatality rate of 83 cases with a known survival outcome was 25.3%.

**Table 1:** Demographic and clinical data of 118 non pregnant adults with listeriosis, Lombardy region, Italy, 2006-2010

**Characteristics**	**No. of patients (%)**
Gender/age	*n* = 118
Female	63 (53.4)
Male	55 (46.6)
≥ 65 years	66 (55.9)
Clinical characteristics	*n* = 103
Underlying condition	91 (88.3)
Solid cancer	30 (29.1)
Immunosuppressive therapy	24 (23.3
Hematologic malignancy	18 (17.5)
Diabetes mellitus	17 (16.5)
Renal insufficiency	16 (15.5)
Liver cirrhosis/chronic	
hepatitis	11 (10.7)
HIV/AIDS	5 (4.8)
Alcoholism	3 (2.9)
Clinical presentation	*n* = 118
Septicemia	90 (76.3)
Meningitis	24 (20.3)
Other infections	4 (3.4)
All-cause fatality rate	*n* = 83
	21 (25.3)

**Figure 1:**
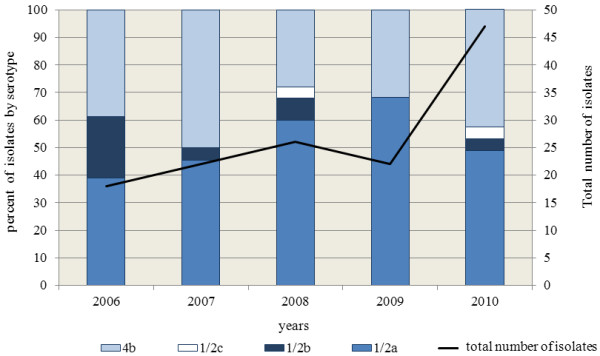
**Annual prevalence of serotypes of *Listeria monocytogenes *in the period under study, Lombardy, Italy, 2006-2010.**

A total of 70 clinical isolates (52.2%) were serotype 1/2a, 52 (38.8%) serotype 4b, 9 (6.7%) serotype 1/2b and three (2.2%) serotype 1/2c. Prevalence of serotypes in the years under study is shown in Figure 1. Between 2006 and 2009, the number of cases caused by serotype 4b was constant, at about 7 to 11 cases per year, but in 2010 it rose up to 20. In contrast, the number of listeriosis cases caused by serotype 1/2a regularly increased from 7 in 2006 to 23 in 2010. The proportion of serotype 1/2a varied between 38.9% and 68.2% and that of serotype 4b between 31.8% and 50.0%.

Pregnancy-associated cases were equally associated with serotype 4b (seven cases) and 1/2a (seven cases), with only one case caused by serotype 1/2b.

The mean age of the non pregnancy-associated cases due to strains of serotype 1/2a was significantly lower than those caused by strains belonging to serotypes 1/2b and 4b (61.4 ± 17.6 vs. 67.2 ± 12.5, P = 0.04). 1/2b and 4b serotypes affected patients >65 years old more frequently than 1/2a (60.9% vs. 49.3%, P = 0.09). The all-cause fatality rate was higher when the *L. monocytogenes* isolates belonged to 1/2b and 4b serotypes than to 1/2a serotype (31.6% vs. 18.6%, P = 0.09).

**Figure 2:**
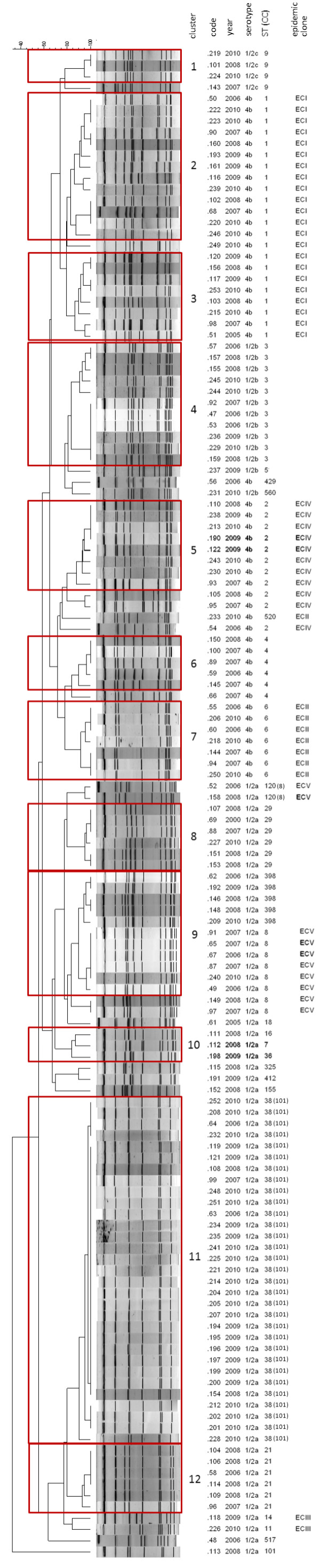
***Asc*I-PFGE restriction patterns of the 134 isolates of *Listeria monocytogenes *identified in Lombardy in the years 2006-2010. **The clonal complex (CC) is indicated in brackets when its identification number is different than the correspondent sequence type (ST).

Seventy-three *Asc*I pulsotypes were recognized among the 134 human isolates. Twelve PFGE clusters, including three to 31 isolates, were identified (Figure 2). Three additional clusters included two isolates. Clusters consistently correlated with serotypes (Figure 2).

**Figure 3:**
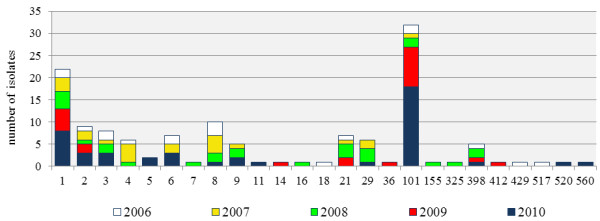
**Distribution of clonal complexes of *Listeria monocytogenes *in the period under study, Lombardy, Italy, 2006-2010.**

The 134 isolates represented 25 STs, which grouped into 23 CCs. Within 4b isolates, three CCs were more prevalent: CC1 (22 isolates), CC2 (11 isolates) and CC3 (11 isolates). CC101 was highly prevalent within the 1/2a serotype (32 isolates). Distribution of CCs in the period 2006-2010 is illustrated in Figure 3.

Table 2 summarizes the clinical and microbiological characteristics of the 94 listeriosis cases included in the PFGE clusters containing more than three isolates. The 15 pregnancy-associated cases were caused by strains belonging to nine PFGE clusters and 13 STs, each including no more than two isolates. Four isolates were unique. Cluster 11 deserves special attention, due to its geographic and temporal size: it included 31 isolates with serotype 1/2a and ST38/CC101. The identification of these isolates began in 2006 (two isolates), continued in 2007 and 2008 (one and two isolates, respectively), and peaked in 2009 and 2010, when 11 and 16 cases of infection were, respectively, caused by these strains. Through the entire 5-year period, cases were distributed in eight out of 12 provinces of Lombardy with the highest prevalence in Bergamo.

**Table 2:** **Clinical and isolate subtype data associated with the major *Asc*I PFGE clusters identified in this study**

**Cluster**	**Gender (no. of cases) M/F**^**a**^	**Age (no. of cases) >65 ys/total**^**a**^	**Clinical data**				**Isolate/subtype data**			
			**Number of pregnancy related cases**	**Infection type**^**a**^		**Number. of cases with underlying condition**^**a,b**^	**No. of isolates**	**Serotype**	**ST/CC**^**c**^	**EC**^**d**^
				**Septicaemia**	**Meningitis**					
2	7/5	8/12	1	9	3	4	13	4b	1	I
3	5/2	3/7	1	3	4	6	8	4b	1	I
4	6/5	5/11	none	10	1	7	11	1/2b	3	
5	5/2	4/7	1	3	4	6	8	4b	2	IV
6	1/3	1/4	2	2	1	3	5	4b	4	
7	1/4	4/5	2	3	2	5	7	4b	6	II
8	2/2	2/4	2	4	none	2	6	1/2a	29	
9	5/3	3/8	3	7	1	6	5	1/2a	398	
							8	1/2a	8	V
11	12/18	26/30	1	20	8	26	31	1/2a	38/101	
12	4/2	1/6	none	4	1	5	6	1/2a	21	

Only the clusters containing more than three isolates are included.

^a ^pregnancy related cases are not included.

^b ^information about underlying diseases was unavailable for some cases.

^c^*ST *sequence type, *CC *clonal complex.

^d^*EC *epidemic clone.

No epidemiological link was detected among patients during the investigations which had been conducted by the local health authorities. More intensive epidemiological investigations following the identification of the increasing number of isolates in 2009 and 2010 failed to show any link with a specific food item.

A total of 53 *L. monocytogenes* isolates (39.5%) tested positive for the EC markers (Figure 2). ECI was the most prevalent (22 isolates 4b/ST1), followed by ECIV (11 isolates 4b/ST2), ECV (eight isolates 1/2a/ST8 and two isolates 1/2a/ST120) and ECII (seven isolates 4b/ST6 and a singleton 4b/ST520). One isolate (233) tested positive for the ECII marker by PCR, but yielded a mixed pattern by MVLST, because the gene *inlB* showed a sequence identical to ECIV, whereas the remaining five genes had a nucleotide sequence consistent with ECII. Only two isolates of serotype 1/2a of ST11 and ST14, respectively, tested positive for ECIII.

## Discussion

In Lombardy, the largest region of Italy with approximately 10 million inhabitants, during 2006-2010 the number of cases of listeriosis reported by the laboratory based surveillance system increased 161% [20]. The detection, identification and reporting procedures in use by the regional laboratories network remained substantially unchanged through the entire period. Consequently, an artifact of surveillance does not appear to explain this increase. Moreover, our findings are consistent with reports of listeriosis in Lombardy by the mandatory notification system of infectious diseases in the same period. Indeed, invasive listeriosis cases notified to the local health authorities increased from 35 in 2006 (incidence rate 0.37 per 100,000 population) to 70 in 2010 (incidence rate 0.74 per 100,000 population) [20]. This increase mainly affected non-pregnant subjects, in accordance with previous reports from other European countries [6-11]. In Italy, in the same period the number of confirmed cases of listeriosis fluctuated between 51 in 2006 and 118 in 2008, but with an incidence rate always < 0.2 cases per 100,000 population [10,20]. Moreover, in 2010 the overall European notification rate was 0.35 cases per 100,000 population with a 3.2% decrease compared with 2009 [10]. The epidemiological trend in Lombardy appears to be counter to that of most European countries in during 2006-2010 [10].

In Lombardy, no common-source outbreaks were notified during the study period to the health authorities. However, the detection of a listeriosis outbreak may be very challenging. Indeed, many concurrent factors, such as the contamination of foods with long shelf lives, the long incubation period, and infrequent infections vs. presumably frequent exposures, may allow a listeriosis outbreak to occur as a succession of apparently unrelated cases [25].

The proportions of patients >65 years and without underlying medical conditions among non pregnancy associated cases were comparable to the rate reported in Europe in 2010 [8-10]. Age, socio-economic factors, differences in diet or food consumption habits and missed epidemic clusters might have confounded our observations. However, it is likely that some strains of *L. monocytogenes* with enhanced virulence may play a role as the etiologic agent in previously healthy persons [26]. This supports the need to refine the field data collection tools for use in epidemiological investigations of listeriosis.

During the study period, the most common *L. monocytogenes* serotypes were 1/2a and 4b. However, unlike serotype 4b, which showed a steady annual prevalence, except for a peak in 2010, the number of serotype 1/2a isolates increased through the entire period. These results agree with previous reports indicating that serotype 1/2a is presumably replacing serotype 4b worldwide as the leading serotype causing human infections [20,27-29].

In our experience, as many as 87% of listeriosis isolates were included in PFGE clusters, a proxy of presumably undetected single source outbreaks. These unrecognized events could have contributed to the increased incidence of listeriosis in Lombardy. A combined use of molecular subtyping and epidemiological investigations, as well as the application of space-time cluster analysis or Geographic Information Systems (GIS), could improve effectiveness of listeriosis outbreaks identification [30].

MLST provided information about distribution of MLST-defined clones in our country. CC1 (4b), CC2 (4b) and CC3 (1/2b), which have been described as prevalent worldwide, were also common in our region [31]. Conversely, CC9 being ranked third in Europe [30], proved to be relatively infrequent among our isolates. In contrast, CC101 (1/2a) was overrepresented in our collection of isolates. In particular, 1/2a/ST38 isolates were recovered from 31 cases of listeriosis occurring through the entire study period, peaking in 2009 and 2010. ST38 is a very infrequent finding in the *Listeria* MLST database at the Pasteur Institute, France, where only three isolates are present from France, 1997, Australia, 2009 and Germany, 2011, respectively.

Moreover, a national database at the Istituto Zooprofilattico Sperimentale of Apulia and Basilicata, including >1000 isolates from human, food and animal source, contains only two isolates belonging to ST38. Of interest, the first one had been isolated in 2005 from a sample of “gorgonzola” cheese, mainly produced in Piedmont and Lombardy. Unfortunately, epidemiological investigations performed by the health authorities were unable to trace a food source. Detection of isolates with identical molecular types in food and clinical cases does not prove *per se* a causal association, unless consistent epidemiological data are available. However, our data strongly suggest the likely occurrence of a prolonged outbreak of foodborne listeriosis which could have been associated with the consumption of regional food products. The likelihood of a listeriosis outbreak lasting many years cannot be disregarded, because of the relationships of *L. monocytogenes* with food and food handling environments, such as its ability to survive in food processing plants and multiply over extended periods of time under harsh chemical and physical conditions [25]. Recently, Knabel *et al.*[18] have reported a 1/2a/CC8 strain as causing listeriosis throughout Canada for at least two decades, with most cases in elderly or immunosuppressed patients and not in pregnant women, as seen in our cluster of 1/2a/ST38 cases.

In a large proportion of our isolates, markers of ECs I to V have been detected. These ECs have been reported to cause multiple outbreaks worldwide and, more recently, to be associated with prevalent STs/CCs [31]. In particular, ECII deserves attention because strains of this clonal group have been responsible for two large multistate outbreaks in 1998-1999 and in 2002 in the United States, but never in Europe [31]. The present findings confirm our previous observations on a more limited sample of isolates from Lombardy and Tuscany regions [17]. Moreover, ECIII has been identified only in two unrelated strains of serotype 1/2a, belonging to STs 11 and 14, respectively. ECIII has been previously associated with ST11 only [31]. ECIV has been detected by MVLST in 11 ST2 isolates. An ECIV strain was previously reported from Italy as the causative agent of an outbreak of febrile gastroenteritis involving 1,566 immunocompetent subjects associated with consumption of a corn and tuna salad in 1997 in Piedmont [32,33]. Eventually, the recently recognized ECV was also identified in 10 isolates into two clusters, comprising eight ST8 and two ST120 isolates, respectively [18]. The frequent detection of EC markers is of concern, as these clones have been associated with nationwide and international outbreaks [20,31]. It has been hypothesized that these epidemic clones have a greater potential for being the cause of foodborne events, perhaps because of their enhanced transmissibility, virulence, and/or persistence in food processing environments [23]. The large proportion of isolates belonging to ECs could inherently confirm the poor sensitivity of traditional surveillance systems in detecting listeriosis outbreaks.

In our study, no specific molecular types could be conclusively associated with maternal-fetal cases, gender, age group or presence/absence of underlying conditions, except for the seven isolates 4b/CC6/ECII belonging to cluster 7, which were all recovered from non pregnant patients younger than 65 years, and the isolates grouped in cluster 11, belonging to serotype 1/2a/CC101, which were more prevalent in females and elderly persons and consistently associated with an underlying condition.

Our study has some limitations. Because of the restricted geographical source of *L. monocytogenes* strains, any generalization of the results is questionable, and an overestimate of clustering has to be considered. The Lombardy notification rate of listeriosis is higher than the overall Italian rate, as previously described. This difference could be explained by a higher sensitivity of the regional surveillance system, as well as by a more frequent exposure to hazardous food sources, such as some soft-ripened cheeses.

## Conclusions

Although molecular epidemiology is not suitable as a stand-alone method, its contribution to epidemiologic investigations and surveillance of *L. monocytogenes* is crucial. Rapid cluster detection can alert authorities to putative outbreaks to be investigated by integrating conventional epidemiological information, increasing the chance to detect and inactivate routes of transmission. Moreover, the proportion of clustered isolates could be used to measure the effectiveness of control and prevention efforts. In the developed countries, the expanding proportion of population subgroups more susceptible to invasive listeriosis along with its severity and high fatality rate argue for improved surveillance, reduced contamination of ready-to-eat foods and enhanced education directed at susceptible populations about avoidance or safe handling of high risk foods.

## Abbreviations

PFGE: Pulsed-field gel electrophoresis; MLST: Multilocus sequence typing; ST: Sequence type; EC: Epidemic clone; MVLST: Multi-virulence-locus sequence typing; CC: Clonal complex.

## Competing interests

The authors declare that they have no competing interests.

## Authors’ contributions

CM, AN and MP conceived the study, participated in its design and coordination and draft the manuscript. AG was in charge of collecting isolates and epidemiological and clinical information and managing the database. AP, AA and CB carried out the phenotypic and molecular subtyping studies. All authors read and approved the final manuscript.

## Pre-publication history

The pre-publication history for this paper can be accessed here:

http://www.biomedcentral.com/1471-2334/13/152/prepub
