# Erratum: Enhanced surveillance of invasive listeriosis in the Lombardy region, Italy, in the years 2006–2010 reveals major clones and an increase in serotype 1/2a

**DOI:** 10.1186/s12879-015-0850-y

**Published:** 2015-05-01

**Authors:** Caterina Mammina, Antonio Parisi, Anna Guaita, Aurora Aleo, Celestino Bonura, Antonino Nastasi, Mirella Pontello

**Affiliations:** Department of Sciences for Health Promotion “G. D’Alessandro”, University of Palermo, Palermo, Italy; Experimental Zooprophylactic Institute of Apulia and Basilicata, Foggia, Italy; Department of Sciences for Health, University of Milan, Milan, Italy; Department of Public Health, University of Florence, Florence, Italy

We published last year a paper [1] about epidemiology of invasive listeriosis in Lombardy according with the data obtained from the enhanced surveillance system which is working in this region since 2006.

Just recently we became aware of a mistake having occurred in the line of Table 1 describing cluster 7. So, we are presenting here the revised Table 1 where it is evident that none of the patients included in cluster 7 was aged >65 years. Likewise, the Discussion contained a wrong sentence about the same Table and, in particular, the data illustrated in the same line as above. We are now confirming that in our study, no specific molecular types could be conclusively associated with maternal-fetal cases, gender, age group or presence/absence of underlying conditions, except for the seven isolates 4b/CC6/ECII belonging to cluster 7 and the isolates grouped in cluster 11. About the isolates belonging to cluster 7 the incorrect text said “which were all recovered from non pregnant patients younger than 65 years”. Now, we are replacing this text with “which were recovered from pregnant women and patients younger than 65 years”.

**Table 1:** **Clinical and isolate subtype data associated with the major** ***Asc*** **I PFGE clusters identified in this study**

**Cluster**	**Gender (no. of cases) M/F** ^**a**^	**Age (no. of cases) >65 ys/total** ^**a**^	**Clinical data**			**Isolate/subtype data**				
			**Number of pregnancy related cases**	**Infection type** ^**a**^		**Number. of cases with underlying condition** ^**a,b**^	**No. of isolates**	**Serotype**	**ST/CC** ^**c**^	**EC** ^**d**^
				**Septicaemia**	**Meningitis**					
2	7/5	8/12	1	9	3	4	13	4b	1	I
3	5/2	3/7	1	3	4	6	8	4b	1	I
4	6/5	5/11	None	10	1	7	11	1/2b	3	
5	5/2	4/7	1	3	4	6	8	4b	2	IV
6	1/3	1/4	2	2	1	3	5	4b	4	
7	1/4	0/5	2	3	2	5	7	4b	6	II
8	2/2	2/4	2	4	0	2	6	1/2a	29	
9	5/3	3/8	3	7	1	6	5	1/2a	398	
							8	1/2a	8	V
11	12/18	26/30	1	20	8	26	31	1/2a	38/101	
12	4/2	1/6	none	4	1	5	6	1/2a	21	

Only the clusters containing more than three isolates are included.

^a^pregnancy related cases are not included.

^b^information about underlying diseases was unavailable for some cases.

^c^ST, sequence type; CC, clonal complex.

^d^EC, epidemic clone.

We regret any inconvenience that our inaccuracies might have caused. We wish to thank Anne Kvistholm Jensen for bringing this issue to our attention.

## References

[1] Mammina C, Parisi A, Guaita A, Aleo A, Bonura C, Nastasi A (2013). Enhanced surveillance of invasive listeriosis in the Lombardy region, Italy, in the years 2006-2010 reveals major clones and an increase in serotype 1/2a. BMC Infect Dis.

